# Exosome-mediated delivery of miR-9 induces cancer-associated fibroblast-like properties in human breast fibroblasts

**DOI:** 10.1038/cddis.2016.224

**Published:** 2016-07-28

**Authors:** S Baroni, S Romero-Cordoba, I Plantamura, M Dugo, E D'Ippolito, A Cataldo, G Cosentino, V Angeloni, A Rossini, M G Daidone, M V Iorio

**Affiliations:** 1 Start Up Unit, Department of Experimental Oncology and Molecular Medicine, Fondazione IRCCS Istituto Nazionale dei Tumori, Amadeo 42 Road, Milan 20133, Italy; 2 INMGEN, Periferico Sur 4809, Arenal Tepepan, Tlalpan, Mexico City 14610, Mexico; 3 Functional Genomics and Bioinformatics, Department of Experimental Oncology and Molecular Medicine, Fondazione IRCCS Istituto Nazionale dei Tumori, Amadeo 42 Road, Milan 20133, Italy; 4 Biomarkers Unit, Department of Experimental Oncology and Molecular Medicine, Fondazione IRCCS Istituto Nazionale dei Tumori, Amadeo 42 Road, Milan 20133, Italy; 5 Unit of Immunotherapy and Anticancer Innovative Therapeutics, Department of Medical Oncology, Fondazione IRCCS Istituto Nazionale dei Tumori, Venezian 1 Road, Milan 20133, Italy

## Abstract

It is established that the interaction between microenvironment and cancer cells has a critical role in tumor development, given the dependence of neoplastic cells on stromal support. However, how this communication promotes the activation of normal (NFs) into cancer-associated fibroblasts (CAFs) is still not well understood. Most microRNA (miRNA) studies focused on tumor cell, but there is increasing evidence of their involvement in reprogramming NFs into CAFs. Here we show that *miR-9*, upregulated in various breast cancer cell lines and identified as pro-metastatic miRNA, affects the properties of human breast fibroblasts, enhancing the switch to CAF phenotype, thus contributing to tumor growth. Expressed at higher levels in primary triple-negative breast CAFs *versus* NFs isolated from patients, *miR-9* improves indeed migration and invasion capabilities when transfected in immortalized NFs; viceversa, these properties are strongly impaired in CAFs upon *miR-9* inhibition. We also demonstrate that tumor-secreted *miR-9* can be transferred via exosomes to recipient NFs and this uptake results in enhanced cell motility. Moreover, we observed that this miRNA is also secreted by fibroblasts and in turn able to alter tumor cell behavior, by modulating its direct target E-cadherin, and NFs themselves. Consistently with the biological effects observed, gene expression profiles of NFs upon transient transfection with *miR-9* show the modulation of genes mainly involved in cell motility and extracellular matrix remodeling pathways. Finally, we were able to confirm the capability of NFs transiently transfected with *miR-9* to promote *in vivo* tumor growth. Taken together, these data provide new insights into the role of *miR-9* as an important player in the cross-talk between cancer cells and stroma.

Tumorigenesis is not considered anymore a tumor cell-autonomous mechanism triggered by accumulation of somatic aberrations, but fostered by a two-way interaction between cancer cells and the surrounding microenvironment.

Cancer cells are indeed integrated in a biologically complex stroma, composed of different cell types (such as immune system components, endothelial cells, fibroblasts and adipocytes) as well as extracellular matrix (ECM), which originates the heterogeneity of the tumor microenvironment (TME).^1^ It is known that a permissive TME has a key role in tumorigenesis.

Fibroblasts, which represent the majority of the stromal cells, are very active in the ECM synthesis, regulation of inflammation and wound healing.^2^ Even though the communication between cancer cells and fibroblasts has been extensively described,^3^ it is still currently unclear how this interaction promotes the activation of quiescent fibroblasts in cancer-associated fibroblasts (CAFs). It has been reported that breast carcinoma-associated stroma differs from its paired normal in deregulated expression of cytokines, ECM molecules and metalloproteinases.^4, 5^

Breast cancer is the leading cause of cancer-related deaths in women.^6^ Clinically, this heterogeneous disease is categorized into four major molecular subtypes: luminal-A, luminal-B, human epidermal growth factor receptor 2 (HER2) overexpressing and triple-negative/basal-like. Triple-negative breast cancer (TNBC) constitutes approximately 15–20% of all breast cancer cases, with the worst outcome of all subtypes.^7^

In breast cancer, the biological characteristics and genetic heterogeneity between CAFs and their paired normal fibroblasts (NFs) have been described.^8, 9^ Breast CAFs are characterized by stronger ability in proliferation and cell motility in comparison with NFs and, consistently with this biological behavior, gene expression profiling showed the abnormal regulation of key signaling pathways as cell adhesion and secreting factors in CAFs.^10^

MicroRNAs (miRNAs) are a class of small non-coding regulatory RNAs that play an important role in various biological processes.^11^ Their extracellular presence as the major RNA component of exosomes suggests an internalization process by TME cells, thus mediating the cancer–host communication and participating in cancer metastasis by adapting the cell niches.^12^ To date, little is known about miRNA expression differences between CAFs and NFs. Array data of primary cultures of CAFs *versus* their paired NFs from resected breast tumor tissues identified 11 dysregulated miRNAs, and their predicted target genes resulted mainly related to adhesion, migration, secretion and cell–cell interaction.^13^ A set of three miRNAs has been described to be involved in reprogramming NFs to CAFs in ovarian cancer^14^ and, very recently, miR-200s were found to contribute to breast cancer cell invasion through CAF activation and ECM remodeling.^15^

In the present work, our attention focused on *miR-9* as a possible player in the cross-talk between breast cancer cells and stroma. Numerous evidence supports this hypothesis: *miR-9* has been described as metastamiR in breast cancer and it resulted markedly upregulated in breast cancer cells compared with normal mammary tissues.^16^ *MiR-9* directly targets E-cadherin (CDH1) leading to increase cancer cell motility and invasiveness.^17^ Even more interestingly, *miR-9* was found to be secreted by different human tumor cell lines, packaged into microvesicles and directly delivered to endothelial cells where it is able to promote migration and neovascularization activating JACK–STAT pathway. These observations suggest that tumor-secreted miRNAs can be involved in intercellular communication.^18^ Moreover, recent data showed that *miR-9* overexpression is associated with epithelial–mesenchymal transition and poor prognosis in breast cancer, leading to its possible use as a biomarker for cancer progression and a target for treatment.^19^

Our data revealed a higher expression of *miR-9* in primary triple-negative breast CAFs *versus* NFs isolated from patients. Cell motility assays of immortalized NFs overexpressing *miR-9* and CAFs where the miRNA was inhibited showed *miR-9's* ability to affect the fibroblast behavior. Furthermore, tumor-secreted *miR-9* can be transferred via exosomes to recipient NFs and this uptake resulted in enhanced cell motility. Gene expression profiles allowed us to identify a subgroup of molecules differentially expressed in NFs overexpressing *miR-9* (NFs/miR-9) mainly involved in cell motility pathways and ECM remodeling. Moreover, *miR-9*-mediated downmodulation of its known target CDH1 in breast cancer cells cultured in conditioned medium from NFs/miR-9 indicated that *miR-9* is also released by fibroblasts and transferred to tumor cells, and provided details regarding the biological mechanisms that could explain both the stronger motility and invasiveness of breast cancer cells observed *in vitro*, and the improved *in vivo* growth following co-injection with NFs/miR-9.

## Results

### *MiR-9* is overexpressed in triple-negative breast CAFs compared with NFs and contributes to acquisition of NFs to a CAF phenotype

**Figure 1:**
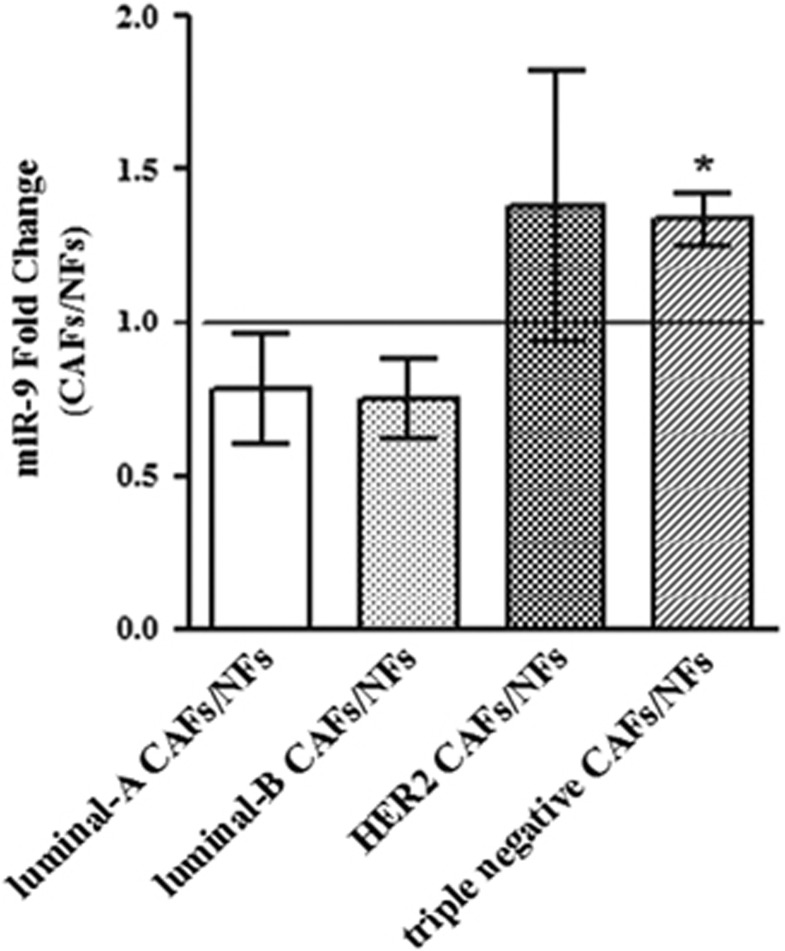
*MiR-9* expression in primary NF/CAF couples. qRT-PCR analysis performed on CAFs and their counterpart NFs isolated from patients affected with different breast cancer subtypes. Data are presented as the mean±S.D. (*P<0.05)

**Figure 2:**
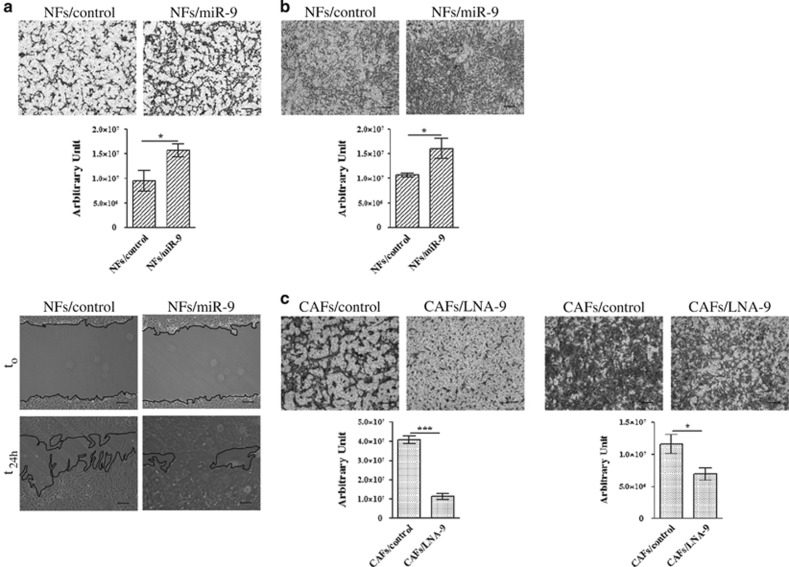
*MiR-9* affects cell motility in NFs and CAFs. (**a**) Migration assays, by transwell (upper panel) and wound healing (lower panel), of NFs after transient transfection with control or *miR-9*. (**b**) Invasion assay of NFs transiently transfected with control or *miR-9*. (**c**) CAF migration (left panel) and invasion (right panel) after transient transfection with control or LNA-9. The migrated or invaded cells are shown by histograms. Data are presented as the mean±S.D. of three views (*P<0.05; ***P<0.0005). Scale bars, 100 *μ*m

To investigate whether a different expression of *miR-9* could play a role in the acquisition of normal fibroblasts to a cancer-associated fibroblast phenotype, the level of mature *miR-9* was first evaluated in couples of primary NFs/CAFs isolated from patients with different breast cancer subtypes (luminal-A, luminal-B, HER2 and triple negative). Interestingly, qRT-PCR analysis revealed a significantly higher level of *miR-9* only in triple-negative CAFs compared with the normal counterpart (Figure 1). To study the functional role of *miR-9*, we decided to use immortalized NFs and CAFs. To verify the purity of the fibroblasts, we tested by western blot analysis the expression of the well-recognized marker *α*SMA, which, as expected, showed expression at a higher level in CAFs (Supplementary Figure S1). Since it was demonstrated that breast CAFs are characterized by stronger cell motility than their paired NFs,^20^ we performed migration, via transwell or wound healing (Figure 2a), and invasion assays (Figure 2b) of NFs transiently transfected with *miR-9* or control (NFs/miR-9 and NFs/control). The overexpression of the miRNA promoted fibroblast motility. Then, in order to clarify if the modulation of *miR-9* also affects CAF properties, the reverse experiment was performed inhibiting miRNA with LNA-9. The transient transfection of CAFs with the inhibitor reduced their migration and invasion compared with control (Figure 2c). These data demonstrate that *miR-9* is involved in the acquisition of a CAF phenotype in breast fibroblasts.

### Tumor-secreted *miR-9* is transferred to NFs via exosomes and increases cell motility

**Figure 3:**
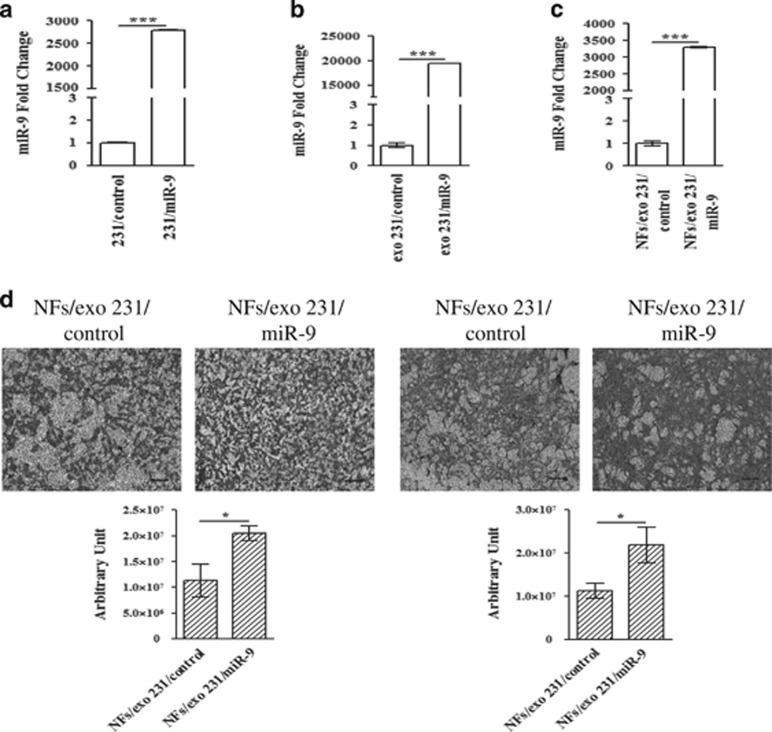
*MiR-9* is delivered to NFs via exosomes and promotes cell motility. (**a** and **b**) qRT-PCR analysis to evaluate *miR-9* level in MDA-MB-231 transiently transfected with control or *miR-9* and in exosomes purified from tumor cell supernatants, respectively. (**c**) MDA-MB-231-secreted exosomes were fed on NFs for 72 h, then RNA was extracted from the recipient cells and analyzed for *miR-9* level by qRT-PCR. The data are shown as normalized relative to *miR-21* (exosomes) or *RNU44* (MDA-MB-231 and NFs, respectively) (***P<0.0005). (**d**) Migration by transwell (left panel) and invasion assays (right panel) of recipient NFs after *miR-9* internalization. Quantitative analysis of the experiments was shown in the lower histograms. Data are presented as the mean±S.D. of three views (*P<0.05). Scale bars, 100 *μ*m

In order to elucidate if tumor-secreted *miR-9* is delivered to the cellular components of the stroma via exosomes, first TNBC MDA-MB-231 cell line was transiently transfected with *miR-9* or control, then the conditioned medium, changed 8 h post-transfection, was collected from transfected cells at 48 h and processed for exosomal purification. *MiR-9* expression was determined by qRT-PCR in transfected cells (Figure 3a) and in the isolated exosomes (Figure 3b) to verify the transfection efficiency and the levels of the miRNA released, respectively. The biochemical characterization of the isolated exosomes revealed the purity of the samples (Supplementary Figure S2). As shown in Figure 3c, the incubation with tumor-secreted exosomes resulted in increase of *miR-9* level in recipient NFs compared to control, thus indicating that the recipient NFs can indeed uptake the exosomes and their cargo. Similar results were obtained with the MDA-MB-468 cell line (Supplementary Figures S3A–C). The biological effect of the exosome-mediated delivery of *miR-9* was investigated performing migration and invasion assays on recipient NFs. The internalization of this miRNA resulted in stronger cell motility (Figure 3d). To confirm that the *miR-9* internalized by NFs was specifically delivered from MDA-MB-231 cancer cells, we repeated the experiment in exosome-deprived medium. No significant difference was detected in *miR-9* transfer to recipient NFs (Supplementary Figures S4A–C). Furthermore, to exclude the presence of micelles, we repeated the same approach introducing an additional ‘medium change step' 24 h post-transfection. The conditioned ‘post-change' medium (pcm) was then collected after additional 24 h and exosomes were isolated. The additional ‘medium change step' did not affect *miR-9* expression in recipient NFs, and led to a similar motility improvement in the presence of *miR-9* containing exosomes (Supplementary Figures S4D and E).

### NFs overexpressing *miR-9* stimulate tumor cell migration by reducing E-cadherin

**Figure 4:**
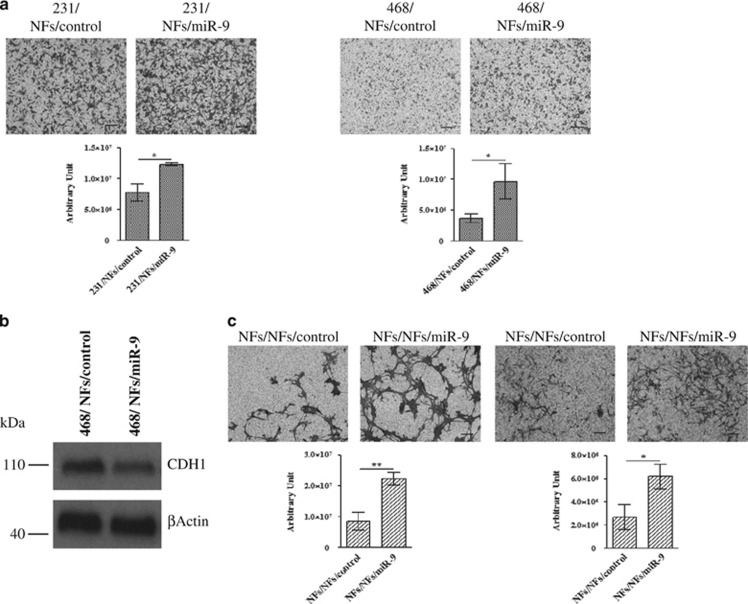
*MiR-9* released by microenvironment to neoplastic cells enhances tumor progression. (**a**) Migration assay of MDA-MB-231 (left panel) and MDA-MB-468 (right panel) co-cultured with conditioned medium derived from NFs transiently transfected with control or *miR-9*. Quantitative analysis of the experiments was shown in the histograms. Data are presented as the mean±S.D. of three views (*P<0.05). (**b**) Western blot analysis of E-cadherin expression in MDA-MB-468 after *miR-9* internalization. (**c**) Migration (left panel) and invasion (right panel) assays of NFs after incubation with conditioned medium from NFs transiently transfected with control or *miR-9*. The migrated or invaded cells are shown by histograms. Data are presented as the mean±S.D. of three views (*P<0.05; **P<0.005). Scale bars, 100 *μ*m

Since we demonstrated that *miR-9* is delivered from breast cancer cells to the microenvironment promoting the neoplastic progression, and considering that the tumor–stroma cross-talk is a two-way communication, we also investigated if the miRNA could be released by fibroblasts to tumor cells. For this reason, co-culture experiments of TNBC MDA-MB-231 and MDA-MB-468 cell lines in conditioned medium derived from NFs transiently transfected with *miR-9* or control were performed. The migration ability of cancer cells was assessed and, as shown in Figure 4a, *miR-9* internalization resulted in stronger motility. *MiR-9* uptake in MDA-MB-231 and MDA-MB-468 was evaluated by qRT-PCR as shown in Supplementary Figure S5. Since it has been reported that co-culture with CAFs induces in tumor cells downregulation of E-cadherin,^21^ known *miR-9* direct target,^17^ we therefore hypothesized that the increase in tumor cell motility induced by *miR-*9 internalization could be explained, at least in MDA-MB-468 cell line, by modulation of this molecule. Indeed, we detected by western blot analysis the downmodulation of E-cadherin protein in MDA-MB-468 grown in contact with the supernatant from NFs overexpressing *miR-9* (Figure 4b). Interestingly, we also observed that the *miR-9* released by NFs/miR-9 induced recipient NFs themselves to enhance migration and invasion (Figure 4c), thus establishing a positive feedback loop. Taken together, these results demonstrated that *miR-9* can be delivered from microenvironment to neoplastic cells, where it is able to enhance tumor progression.

### Identification of differentially expressed genes in NFs upon *miR-9* transfection

**Figure 5:**
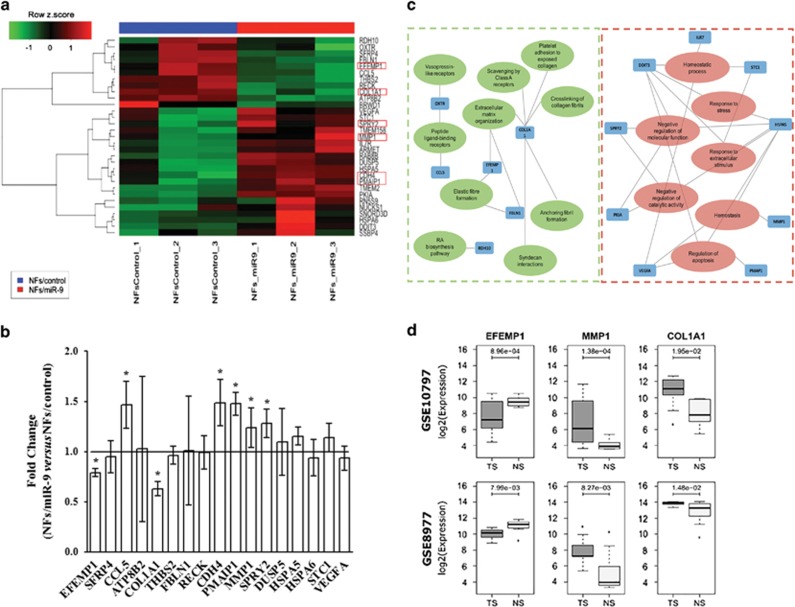
Differentially expressed genes in NFs overexpressing *miR-9*. (**a**) Hierarchical clustering analysis of *miR-9* exogenous expressing in NFs. Heatmap: rows correspond to differentially expressed genes and columns to samples. Red represents elevated and green downmodulated expression. (**b**) Validation through qRT-PCR analysis of the differentially expressed genes related with cell motility and ECM organization. The relative expression levels are shown as fold change of NFs/miR-9 *versus* NFs/control. (**c**) Interaction network of the significantly enriched gene ontologies and pathways of the differentially expressed genes in the *miR-9* transient transfection model. Green and red edges represent the down- or upmodulated pathways, respectively, according to the expression of the connected genes (blue node). (**d**) Boxplots showing the expression levels of the three selected genes in two public gene expression data sets of tumor (TS) and normal stroma (NS) from human breast specimens. *P*-values from two-tailed Student's *t*-test are reported

To clarify the molecular alterations triggered by *miR-9* to induce the acquisition of breast NFs to a CAF phenotype, gene expression profile of NFs transiently transfected with *miR-9* or control was performed. We identified 11 downregulated and 20 upregulated genes in NFs overexpressing *miR-9* compared with NFs/control based on a minimum log2 fold change of 0.7 and *P*<0.05 (Figure 5a). We selected 17 genes (8 downregulated and 9 upregulated) related with cell motility pathways and ECM remodeling to be validated by qRT-PCR in NFs/control *versus* NFs/miR-9 (Figure 5b). Downregulated genes encode for proteins mainly involved in ECM organization, whereas upmodulated molecules are involved in the regulation of apoptosis and response to extracellular stimuli (Figure 5c). EGF-containing fibulin-like extracellular matrix protein 1, collagen type 1 alpha 1, sprouty homolog 2, matrix metalloproteinase-1, retinal cadherin, phorbol-12-myristate 13-acetate (EFEMP1, COL1A1, SPRY2, MMP1, CDH4 and PMAIP1, respectively) showed significant differential expression, consistently with the microarray analysis.

To assess whether the deregulation of our selected genes was detectable in human clinical specimens, we analyzed public gene expression data of epithelial and stromal cells from breast cancer patients compared with their normal counterparts. We decided to test the expression status of EFEMP1, COL1A1 and MMP1, assuming their relevance in the pathways of our interest. In data set GSE10797 (Figure 5d), we observed significant downregulation of EFEMP1 and upregulation of MMP1 in stromal cells of 28 breast cancer patients compared with stromal cells from 5 normal individuals who received reduction mammoplastic surgery. These results were consistent with the modulation observed in gene expression data of NFs transfected with *miR-9* compared with control. On the contrary, COL1A1 displayed an opposite behavior if compared with our gene profiling results. Moreover, the same gene expression patterns were observed in the epithelial cells from the same patients, suggesting that these genes play their role in both cell types (Supplementary Figure S6). In a second gene expression data set of 7 breast tumor and 15 normal stroma samples (GSE8977; Figure 5d), we observed the same differential expression for EFEMP1 and MMP1. Again, COL1A1 resulted differentially expressed but with an opposite behavior in comparison with *in vitro* data. Taken together, these results show that some of the transcriptional alterations identified in NFs after transient transfection with *miR-9* are also detected in stroma of breast cancer patients.

### NFs overexpressing *miR-9* promote *in vivo* tumor growth

**Figure 6:**
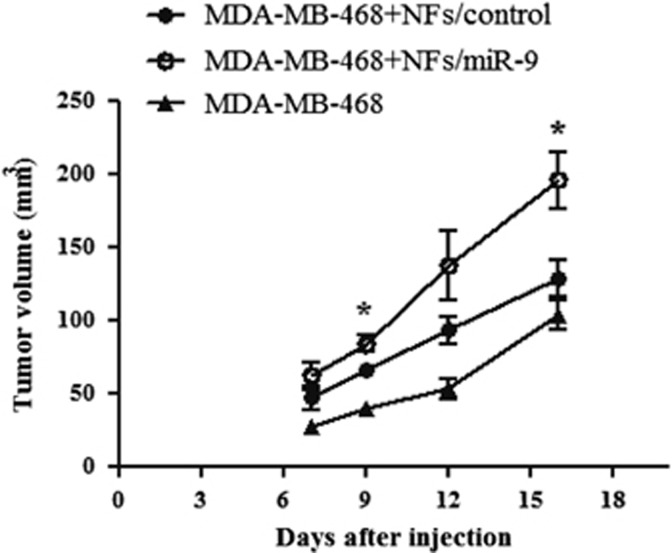
NFs overexpressing *miR-9* promote *in vivo* tumor growth. Evaluation of tumor volumes in SCID mice co-injected with MDA-MB-468 cells and NFs transiently transfected with control or *miR-9*. The control group was injected with MDA-MB-468. Data are presented as the mean±S.D. (*n*=6; *P<0.05)

Several studies revealed that the conversion of NFs into CAFs may occur at the initiation phase of breast cancer, inducing malignant transformation of adjacent mammary epithelial cells.^8^ Our results show that the exosome-vehicolated-*miR-9* released from transfected fibroblasts promoted tumor cell aggressiveness *in vitro*, modulating genes involved in cell motility and ECM remodeling. To confirm the capability of *miR-9* overexpressing NFs to affect cancer progression, we monitored *in vivo* tumor growth of MDA-MB-468 cells co-injected with NFs/miR-9 or control in the mammary fat pad of SCID mice (6 mice for group). Moreover, a control group of six mice was injected with parental MDA-MB-468 cells to evaluate tumor development and progression. We measured tumor volumes for 2 weeks and, as shown in Figure 6, tumor growth was significantly increased in mice co-injected with MDA-MB-468 cells and NFs transiently transfected with *miR-9* compared with the control group. In conclusion, these data confirm that a higher expression of *miR-9* in the TME plays an important role in breast cancer progression.

## Discussion

It is well supported that miRNAs are involved in the progression of cancer, acting as tumor suppressors as well as oncogenes depending on the target molecules; however, their activity in the tumor stroma needs to be further investigated.^22^ Emerging reports of miRNA abilities in reprogramming normal into cancer-associated fibroblasts have been described.^14, 23^ MiRNA microarrays of CAFs and NFs in breast cancer identified a small group of differentially expressed miRNAs,^13^ including miR-200 s, which have been recently demonstrated to be direct mediators of NFs reprogramming into CAFs and ECM remodeling.^15^

In the present work, we show that *miR-9* acts as an important player in the communication between breast cancer cells and the cellular component of the TME and it is able to promote the conversion of NFs toward a CAF-like phenotype.

Zhao and colleagues did not report *miR-9* as deregulated in breast CAF/NF couples obtained from patients; however, we observed a significantly higher level of this miRNA in primary triple-negative CAFs compared with the normal counterpart. This first evidence is consistent with the association of *miR-9* with aggressive breast cancer phenotype^19^ and with our own data (unpublished).

In line with the genetic heterogeneity between breast CAFs and NFs,^20, 24^ our expression profile identified in NFs overexpressing *miR-*9 a signature of differentially expressed genes correlated with cell motility and ECM organization: specifically members of matrix metalloproteinases, fibulins and collagens. MMPs are multifunctional enzymes capable of cleaving the ECM components, growth factors, cytokines, cell surface-associated adhesion and signaling receptors.^25^ In particular, we observed the up-modulation of MMP1, which is reported to be highly expressed in poor-outcome breast carcinomas^26^ and associated with breast tumor progression.^27^ Downmodulation of EFEMP1, a member of fibulin family, secreted proteins associated with the ECM scaffold and regulators of cell proliferation and migration, which is consistent with the reduction observed in a cohort of sporadic breast cancer^28^ and the recently demonstrated decrease during breast tumor progression.^29^ Intriguingly, we also detected the downregulation of COL1A1, the major structural component of ECM which drives the fundamental physiological processes that allow to adapt the microenvironment to changing functional demands, and which is reported as a direct target of MMP1 activity.^30^ The degradation of type I collagen by MMP1 was indeed shown to be associated with rapid progression, poor overall survival and secondary metastasis, and it appears that this process may have a pivotal role in the acquisition of invasive characteristics in breast cancer.^31^ Whereas the *in silico* analysis of EFEMP1 and MMP1 expression in human series confirmed that the same modulations are also detected in stromal component of breast cancer patients, COL1A1 showed an opposite behavior. This discordance could be explained by the heterogeneity of stromal tissue, which contains immune and endothelial cells beside fibroblasts and by the different breast cancer subtypes of patients analyzed.

Our data demonstrated that the modulation of gene expression profile and the acquisition of a CAF-like phenotype in recipient fibroblasts can be induced by tumor cells through exosome-mediated delivery of *miR-9*. This is not surprising, since circulating miRNAs seem to be mainly associated to exosomes, and exchanged between different cell types as a communication tool.^32^ Moreover, tumor-secreted *miR-9* has been demonstrated to affect also endothelial cell proliferation,^18^ thus suggesting that this miRNA is probably exploited by tumor cells as a sort of ‘signal' to convert the microenvironment into a pro-tumoral niche.

Even more interestingly, we demonstrated the existence of a positive circuitry, where ‘converted' fibroblasts are in turn able to promote tumor growth and aggressiveness: our results revealed that conditioned medium derived from NFs overexpressing *miR-9* increased the aggressiveness of triple-negative breast cancer MDA-MB-231 and MDA-MB-468 cell lines, consistent with the well-established role of CAFs in promoting cancer cell progression.^20^ We demonstrated that this stronger cell capability could be caused, at least in MDA-MB-468, by the reduction of the E-cadherin, calcium-dependent cell–cell adhesion glycoprotein that has been demonstrated to be a direct target of *miR-9*.^17^ In MDA-MB-231, where E-cadherin is epigenetically silenced, other molecules are probably regulated by *miR-9* in order to obtain the observed biological effect.

Multiple studies focused on the cancer invasion- and progression-promoting role of breast CAFs. In contrast to NFs, mammary CAFs induce local invasion of primary tumor cells trough epithelial–mesenchymal transition and ECM remodeling^33^ and co-implantation tumor xenograft models demonstrated that CAFs from human breast cancer significantly induce tumor growth than NFs from the same patients.^34^ Even though the mechanisms by which NFs are converted into CAFs are still unclear, here we show that the overexpression of *miR-9* in normal fibroblasts was sufficient to increase tumor growth in mouse models, corroborating the capability of this miRNA to reprogram NFs into CAFs, thus promoting tumor initiation and progression.

In conclusion, the involvement of *miR-9* in reprogramming the microenvironment, activating tumor-promoting abilities in normal fibroblasts, as migration and invasion, in addition to its tumor-intrinsic pro-metastatic role, confers to this miRNA a relevant potential as a therapeutic target in breast cancer.

## Materials and Methods

### Isolation of primary fibroblasts and cell culture

Primary NFs and CAFs were isolated from specimens belonging to patients who underwent surgery at Fondazione IRCCS Istituto Nazionale dei Tumori of Milan (INT) and who signed an informed consent to donate the leftover tissue after diagnosis to INT for research. Surgically resected tumor and normal tissues were sampled by pathologists immediately after surgery and then enzymatically digested with Collagenase/Hyaluronidase (Stem Cell Technologies, Vancouver, BC, Canada) overnight at 37 °C in agitation. After filtration through a 100-*μ*m pore filter (Millipore, Billerica, MA, USA), cells were plated using fibroblast growth in FGM-2 medium (Lonza, Walkersville, MD, USA). Immortalized NFs and CAFs^34^ were cultured in FGM-2 medium. TNBC MDA-MB-231 cell line was cultured in RPMI 1610-medium (Euroclone, Milan, Italy) with 10% fetal bovine serum (FBS) (Thermo Fisher Scientific, Waltham, MA, USA), MDA-MB-468 cell line was cultured in DMEM (Euroclone) with 10% FBS. Breast cancer cell lines were purchased from ATCC (Rockville, MD, USA). All cells were maintained at 37 °C under 5% CO_2_.

### RNA extraction and quantitative real-time PCR

Total RNA was isolated using QIAzolLysis Reagent (Qiagen Sciences, Germantown, MD, USA) according to the manufacturer's instruction. The purified RNA was subjected to reverse transcription reactions by using SuperScript III Reverse Transcriptase (Thermo Fisher Scientific) or TaqManMicroRNA Reverse Transcription kit (Thermo Fisher Scientific). qRT-PCR with Fast SYBRGreen Master Mix (Thermo Fisher Scientific) was used to evaluate the expression of the genes and GAPDH as an internal control. All the primer sequences are reported in Table 1. MicroRNA expression levels were evaluated by qRT-PCR performed with TaqMan Fast Universal PCR Master Mix (Thermo Fisher Scientific). *Mir-21* or *RNU44* (Thermo Fisher Scientific) was used as an internal control. qRT-PCR assays were performed in StepONEPlus Real-Time PCR system (Thermo Fisher Scientific) and the relative expression was calculated using the comparative 2^−^^ΔCt^ method.

### Mimics and inhibitors

*MiR-9* precursor and negative control were purchased as Pre-miR precursor molecules (Thermo Fisher Scientific). Locked nucleic acid (LNA) against *miR-9* and the corresponding control were purchased from EXIQON (Vedbaek, Denmark). Briefly, fibroblasts and breast cancer cells were transfected for 24 or 48 h, respectively, with 25 nM miRNA precursor or inhibitor using Lipofectamine 2000 (Thermo Fisher Scientific) according to the manufacturer's instruction.

### Preparation of conditioned medium and exosome extraction

The media from breast cancer cells and fibroblasts were collected and centrifuged at 3000 r.p.m. for 10 min and the supernatants resulted as conditioned media. Cancer-secreted exosomes were extracted using the ExoQuick-TCExosome Precipitation Solution (System Biosciences, Mountain View, CA, USA). Briefly, the appropriate volume of ExoQuick-TCExosome Precipitation Solution was added to the breast cancer conditioned medium and refrigerated overnight. The sample was first centrifuged at 1500 r.p.m. for 30 min at 4 °C and then at 3000 r.p.m. for 5 min. The exosome pellet was resuspended in 250–500 *μ*l of appropriate cell medium and incubated 72 h with recipient cells. Exosome-deprivated medium was obtained upon 25 000 r.p.m. ultracentrifugation for 90 min.

### Migration, wound healing and invasion assays

Cell migration and invasion were performed in Transwell Permeable Support 8.0 *μ*m (Corning, Kennebunk, ME, USA). Briefly, 1.5 × 10^5^ cells in 300 *μ*l of serum-free medium were seeded in the top of the chamber, for invasion experiments in the presence of Matrigel (Corning). Medium supplemented with 10% FBS was used as a chemoattractant in the bottom chamber. After 6, 12 or 24 h of incubation at 37 °C the migrating/invading cells on the opposite side of the filter were fixed with cold ethanol and stained with 0.4% Sulforhodamine B (GE Healthcare Life Sciences, Little Chalfont, UK), then counted with the Image-Pro Plus 7.0 software (Media Cybernetics, Rockville, MD, USA). For wound-healing assays, 3.0 × 10^5^ fibroblasts were seeded in six-well plates. An artificial gap was created on the confluent cell monolayer with a plastic tip and the images of the wound area were captured in the following 24 h.

### Protein extraction and western blot analysis

Whole-cell lysates and total exosomal proteins were prepared using RIPA buffer (50 mM Tris-HCl pH 7.4, 150 mM NaCl, 1% NP40, 0.5% sodium deoxycholate, 0.1% sodium dodecyl sulfate). Fifteen micrograms total proteins were electrophoretically separated on NuPAGE 4–12% Bis-Tris Gel (Thermo Fisher Scientific). Western blot analyses were performed with primary antibodies: anti-*α*SMA, anti-β-actin peroxidase-linked, anti-Vinculin, anti-*α*Tubulin (1 : 500; 1 : 10 000 and 1 : 1000, respectively; Sigma-Aldrich, St. Louis, MO, USA), anti-CDH1, anti-Rab5B (1 : 1000; Santa Cruz Biotechnology, Dallas, TX, USA), anti-Flot1 (1 : 1000; Cell Signalling, Boston, MA, USA), anti-Lamp2, anti-CD63, anti-COL1A1 (1 : 1000, BD Biosciences, Franklin Lakes, NJ, USA) and the corresponding secondary antibodies anti-mouse and anti-rabbit peroxidase-linked (1 : 10 000; GE Healthcare Life Sciences). The signals were visualized by ECL Prime Western Blotting Detection Reagent (GE Healthcare Life Sciences).

### Gene expression profiling and analysis of public data sets

Total RNA derived from three independent biological samples of immortalized NFs transiently transfected with control (NFs/control) or *miR-9* (NFs/miR-9) was isolated using QIAzolLysis Reagent according to the manufacturer's instruction. RNA quantity was spectrophotometrically determined and the quality was evaluated by capillary electrophoresis (Agilent 2100 Bioanalyzer; Agilent Technologies, Santa Clara, CA, USA). Only the samples with RNA Integrity Number greater than 8.0 were further processed for microarray analysis. For gene expression profiling, 800 ng of total RNA was reverse transcribed, biotin-labeled and amplified using Illumina TotalPrepRNA Amplification kit (Thermo Fisher Scientific). One microgram of each cRNA amplified sample was added to Hyb E1 hybridization buffer containing 37.5% (w/w) formamide and hybridized to array HumanHT-12 v4 Expression BeadChip (Illumina Inc., San Diego, CA, USA) at 58 °C for 18 h. Arrays were washed and stained using 1 *μ*g/ml of Cy3-streptavidin (GE Healthcare Life Sciences). Image files were quantified in Illumina BeadStudio version 3.3.8 and raw data were analyzed (variance stabilizing transformation, log2 transformation and quantile normalization) in R statistical environment using the lumi package^35^ implemented in Bioconductor. To reduce the non-biological experimental variation or batch effects we applied ComBat adjustment method^36^ to the normalized data. In all, 28 668 mRNAs were evaluated with HumanHT-12 V4 (Illumina). Gene expression data were deposited in the Gene Expression Omnibus data repository (GEO) with accession number GSE76996. To identify differentially expressed genes between NFs/miR-9 and NFs/control, a moderate *t*-test was performed using limma package.^37^ Significant genes were selected based on a minimum log2 fold change of 0.7 and *P*<0.05. To define a significantly enrichment of Gene Ontology and pathways of the differentially expressed genes in the *miR-9* transitory transfection model, the DAVID annotation chart tool (https://david.ncifcrf.gov/summary.jsp) and Reactome tool (http://www.reactome.org) were used and analyzed in Cytoscape. The significantly enrichment processes were defined based on the *P*<0.05. For public gene expression data of stromal breast tissues raw CEL files were downloaded from GEO with accession numbers GSE10797 and GSE8977. Raw data were processed using the frozen robust multi-array average (frma) method^38^ as implemented in the ‘frma' package of Bioconductor. ProbeSets annotation was retrieved from the hgu133plus2.db package and for probesets mapping on the same gene the one with highest mean expression across samples was selected. Differential expression of selected genes was assessed by two-tailed Student's *t*-test and a *P*-value <0.05 was considered for statistical significance.

### Tumor growth analysis in orthotopic xenografts

All animal experiments were approved by the Ethics Committee for the Animal Experimentation of Fondazione IRCCS Istituto Nazionale dei Tumori of Milan. Immortalized NFs (5.0 × 10^6^ cells/mouse) were transiently transfected with *miR-9* precursor or control for 24 h and co-injected with TNBC MDA-MB-468 (5.0 × 10^6^ cells/mouse) in the mammary fat pad of 8-week-old female SCID mice (Charles River, Wilmington, MA, USA). Cells were resuspended in 200 *μ*l final volume of FGM-2 medium at a ratio 1 : 1 with Matrigel. Tumor growth was monitored every 3–4 days until the time of killing and the volume (*V*_T_) was calculated according with the formula: *V*_T_=(*S*^2^ × *L*)/2, where *S* and *L* correspond with the short and long dimension, respectively. Tumors were harvested after 16 days from the inocule. Statistical significance was analyzed by the two-tailed Student's *t*-test and a *P*-value of less than 0.05 was considered significant.

## Figures and Tables

**Table 1:** Quantitative RT-PCR primers

**Gene ID**	**Primer**	**Sequence**
EFEMP1	Forward	ATTGCCACCAAAGATGCGTG
	Reverse	GCTGCCAATTGAAACCCAGG
SFRP4	Forward	GGCGCACCAGTCGTAGTAAT
	Reverse	TCTTGGGACTGGCTGGTTTG
CCL5	Forward	CGTGCCCACATCAAGGAGTA
	Reverse	TCGGGTGACAAAGACGACTG
ATP8B2	Forward	ACCTTGAGAGCTGTTCCCCTT
	Reverse	ATCTCACCCAGCAAGATCCC
COL1A1	Forward	GTGGCCTGCCTGGTGAG
	Reverse	GCACCATCATTTCCACGAGC
THBS2	Forward	CAGACCGACGTGGACAATGA
	Reverse	GTGGCCGTCGTCATCTATGT
FBLN1	Forward	TGCCATGAGAATCGGGAGTG
	Reverse	GCTTGGATGTTGGTGGGGAA
RECK	Forward	TGATGTATGTGAACAGATTTTCTCC
	Reverse	TGGGCAATAATCTGGGGCTC
CDH4	Forward	AGGCTGGGTTCTCTGAAGATG
	Reverse	ATATTGTGTCCCCTTGGTCCC
PMAIP1	Forward	GGAAGTCGAGTGTGCTACTCA
	Reverse	TCCTGAGCAGAAGAGTTTGGA
MMP1	Forward	ACAGCTTCCCAGCGACTCTA
	Reverse	GGGCCACTATTTCTCCGCTT
SPRY2	Forward	TCAGAGCCATCCGAAACACC
	Reverse	TCGTGTTTGTGCTGAGTGGA
DUSP5	Forward	ACAGCCCTGCTGAATGTCTC
	Reverse	GGAGCTAATGTCAGCCGTGT
HSPA5	Forward	TCTTGTTGGTGGCTCGACTC
	Reverse	ATCTGGGTTTATGCCACGGG
HSPA6	Forward	CTGCCAAAAACTCGCTGGAG
	Reverse	GCAAGGACTTCCCGACACTT
STC1	Forward	CACCCACGAGCTGACTTCAA
	Reverse	GGGATGTGCGTTTGATGTGG
*α*SMA	Forward	CATCACCAACTGGGACGACATGGAA
	Reverse	GCATAGCCCTCATAGATGGGGACATTG
FAP	Forward	TGCCACCTCTGCTGTGC
	Reverse	GAAGCATTCACACTTTTCATGGT
SDF1	Forward	TGAGAGCTCGCTTTGAGTGA
	Reverse	CACCAGGACCTTCTGTGGAT
GAPDH	Forward	GCTGGCGCTGAGTACGTCGTGGAGT
	Reverse	CACAGTCTTCTGGGTGGCAGTGATGG

Abbreviation: *α*-SMA, alpha-smooth muscle actin; ATP8B2, ATPase aminophospholipid transporter type 8B member 2; CCL5, chemokine ligand 5; CDH4, retinal cadherin; COL1A1, collagen type 1 alpha 1; DUSP5, dual specificity phosphatase 5; EFEMP1, epidermal growth factor-containing fibulin-like extracellular matrix protein 1; FAP, fibroblast activation protein; FBLN1, fibulin 1; GAPDH, glyceraldehyde 3-phosphate dehydrogenase; HSPA5, heat-shock protein 5; HSPA6, heat-shock protein 6; MMP1, matrix metalloproteinase-1; PMAIP1, phorbol-12-myristate 13-acetate induced protein 1; RECK, reversion inducing cysteine-rich protein with kazal motifs; SFRP4, secreted frizzled-related protein 4; SPRY2, sprout homolog 2; STC1, stanniocalcin 1; THBS2, thrombospondin 2.

## Abbreviations

NFs: normal fibroblasts
CAFs: cancer-associated fibroblasts
microRNA: miRNA
ECM: extracellular matrix
TME: tumor microenvironment
HER2: human epidermal growth factor receptor 2
TNBC: triple-negative breast cancer
CDH1: E-cadherin
EFEMP1: epidermal growth factor-containing fibulin-like extracellular matrix protein 1
SFRP4: secreted frizzled-related protein 4
CCL5: chemokine ligand 5
ATP8B2: ATPase aminophospholipid transporter type 8B member 2
COL1A1: collagen type 1 alpha 1
THBS2: thrombospondin 2
FBLN1: fibulin 1
RECK: reversion inducing cysteine-rich protein with kazal motifs
CDH4: retinal cadherin
PMAIP1: phorbol-12-myristate 13-acetate induced protein 1
MMP1: matrix metalloproteinase-1
SPRY2: sprout homolog 2
DUSP5: dual specificity phosphatase 5
HSPA5: heat-shock protein 5
HSPA6: heat-shock protein 6
STC1: stanniocalcin 1
*α*-SMA: alpha-smooth muscle actin
FAP: fibroblast activation protein
GAPDH: glyceraldehyde 3-phosphate dehydrogenase.
