# Correction: Exosome-mediated delivery of miR-9 induces cancer-associated fibroblast-like properties in human breast fibroblasts

**DOI:** 10.1038/s41419-025-07382-w

**Published:** 2025-03-11

**Authors:** Sara Baroni, S. Romero-Cordoba, I. Plantamura, M. Dugo, E. D’Ippolito, A. Cataldo, G. Cosentino, V. Angeloni, A. Rossini, M. G. Daidone, M. VIorio

**Affiliations:** 1 Start Up Unit, Department of Experimental Oncology and Molecular Medicine, Fondazione IRCCS Istituto Nazionale dei Tumori, Amadeo 42 Road, Milan, 20133 Italy; 2 INMGEN, Periferico Sur 4809, Arenal Tepepan, Tlalpan, Mexico City, 14610 Mexico; 3 Functional Genomics and Bioinformatics, Department of Experimental Oncology and Molecular Medicine, Fondazione IRCCS Istituto Nazionale dei Tumori, Amadeo 42 Road, Milan, 20133 Italy; 4 Biomarkers Unit, Department of Experimental Oncology and Molecular Medicine, Fondazione IRCCS Istituto Nazionale dei Tumori, Amadeo 42 Road, Milan, 20133 Italy; 5 Unit of Immunotherapy and Anticancer Innovative Therapeutics, Department of Medical Oncology, Fondazione IRCCS Istituto Nazionale dei Tumori, Venezian 1 Road, Milan, 20133 Italy

Correction to: *Cell Death and Disease* 10.1038/cddis.2016.224, published online 28 July 2016

In this article the author name “S Baroni” has been updated to “Sara Baroni”.

The original article has been updated.

