# Supplementary figures and images for: Exosome-mediated delivery of miR-9 induces cancer-associated fibroblast-like properties in human breast fibroblasts
Source: Cell Death Dis. 2016 Jul 28;7(7):e2312–. doi: 10.1038/cddis.2016.224 (PMC4973361; doi:10.1038/cddis.2016.224)

## Slide 1
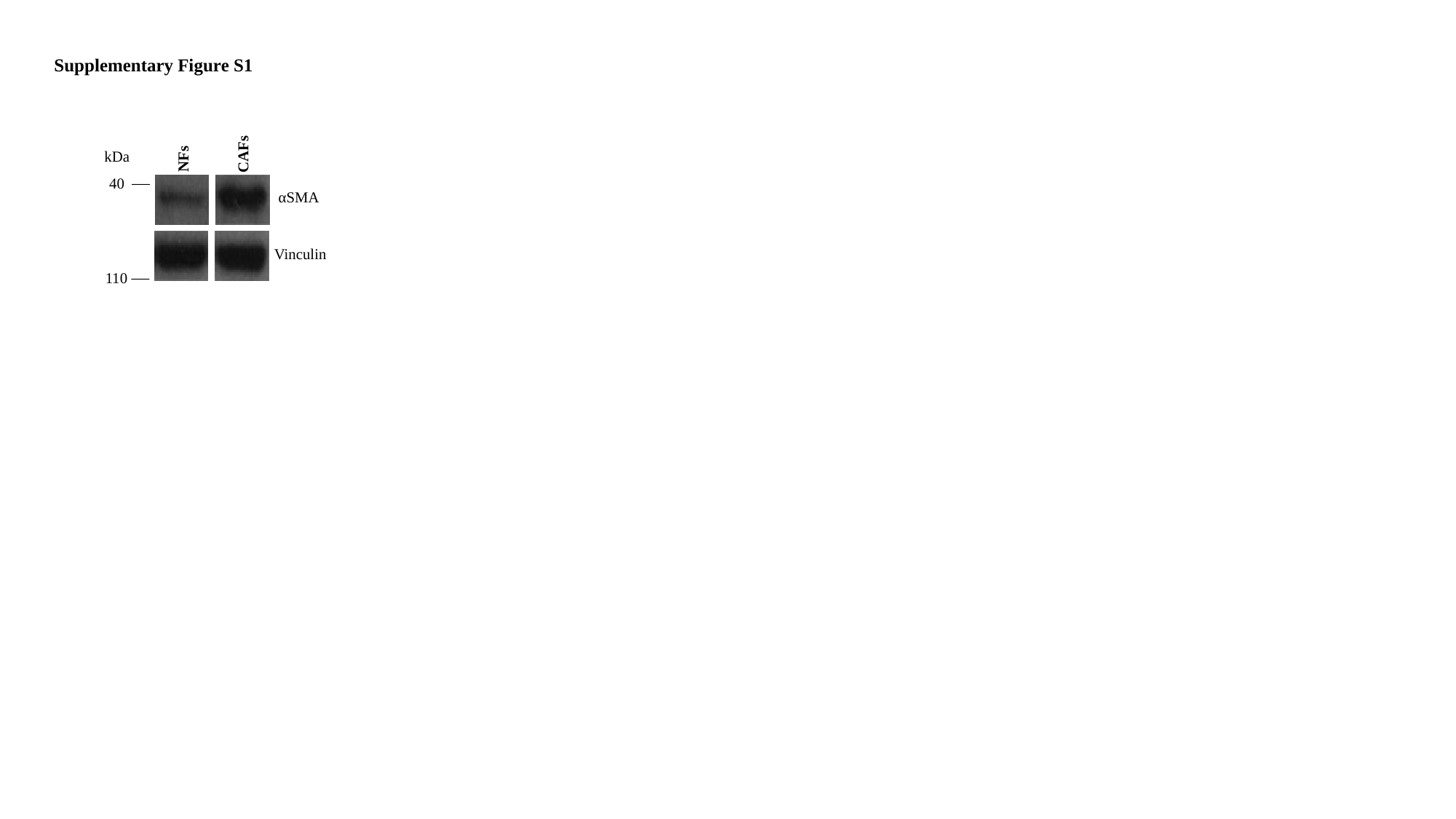

Supplementary Figure S1
CAFs
kDa
 NFs
40
αSMA
Vinculin
110

Supplement: Supplementary Figure S1 [file cddis2016224x1.ppt]

## Slide 1
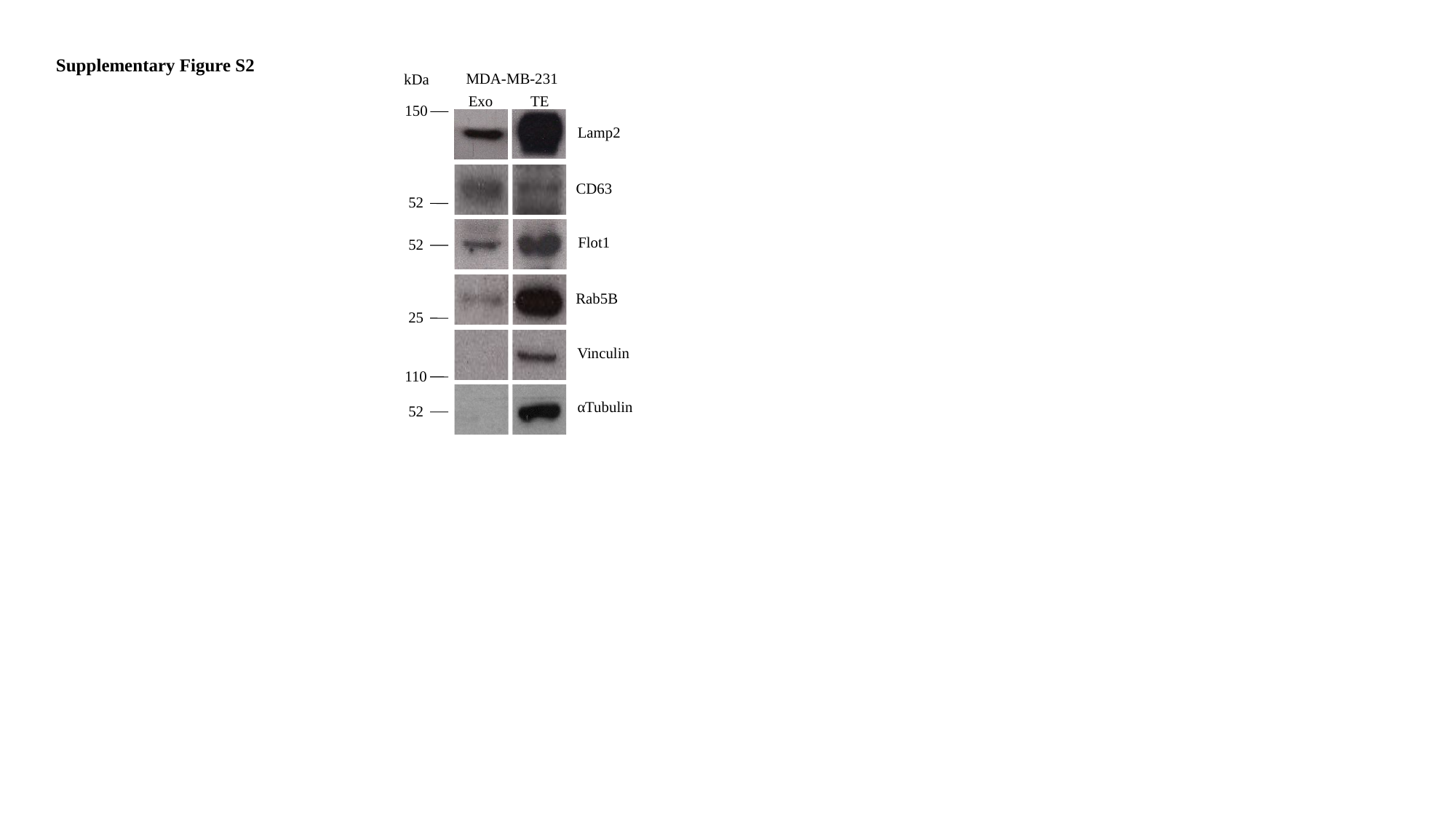

Supplementary Figure S2
MDA-MB-231
kDa
Exo
TE
150
Lamp2
CD63
52
Flot1
52
Rab5B
25
Vinculin
110
αTubulin
52

Supplement: Supplementary Figure S2 [file cddis2016224x2.ppt]

## Slide 1
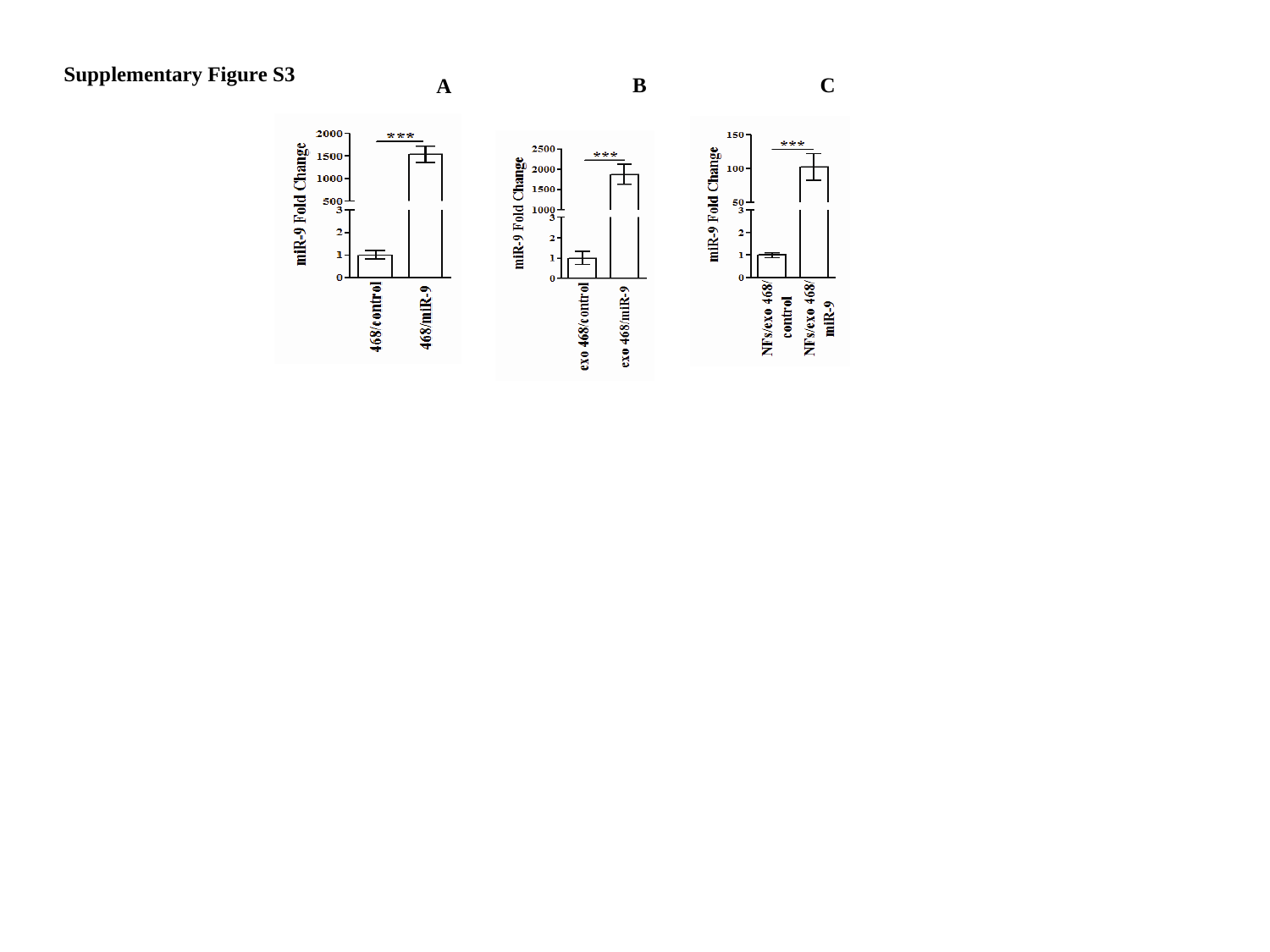

Supplementary Figure S3
B
C
A

Supplement: Supplementary Figure S3 [file cddis2016224x3.ppt]

## Slide 1
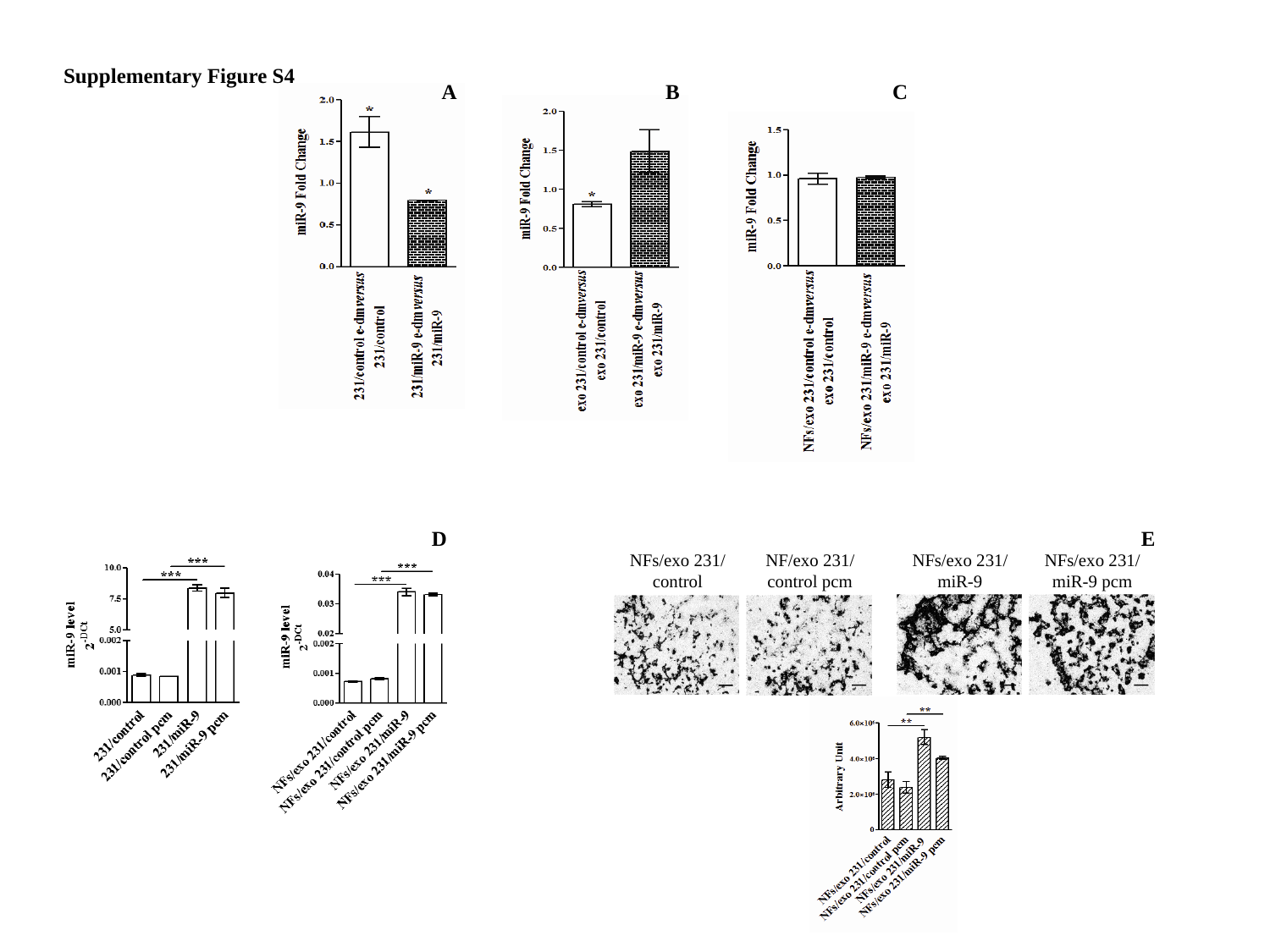

Supplementary Figure S4
B
C
A
D
E
NFs/exo 231/
miR-9 pcm
NF/exo 231/
control pcm
NFs/exo 231/
miR-9
NFs/exo 231/
control

Supplement: Supplementary Figure S4 [file cddis2016224x4.ppt]

## Slide 1
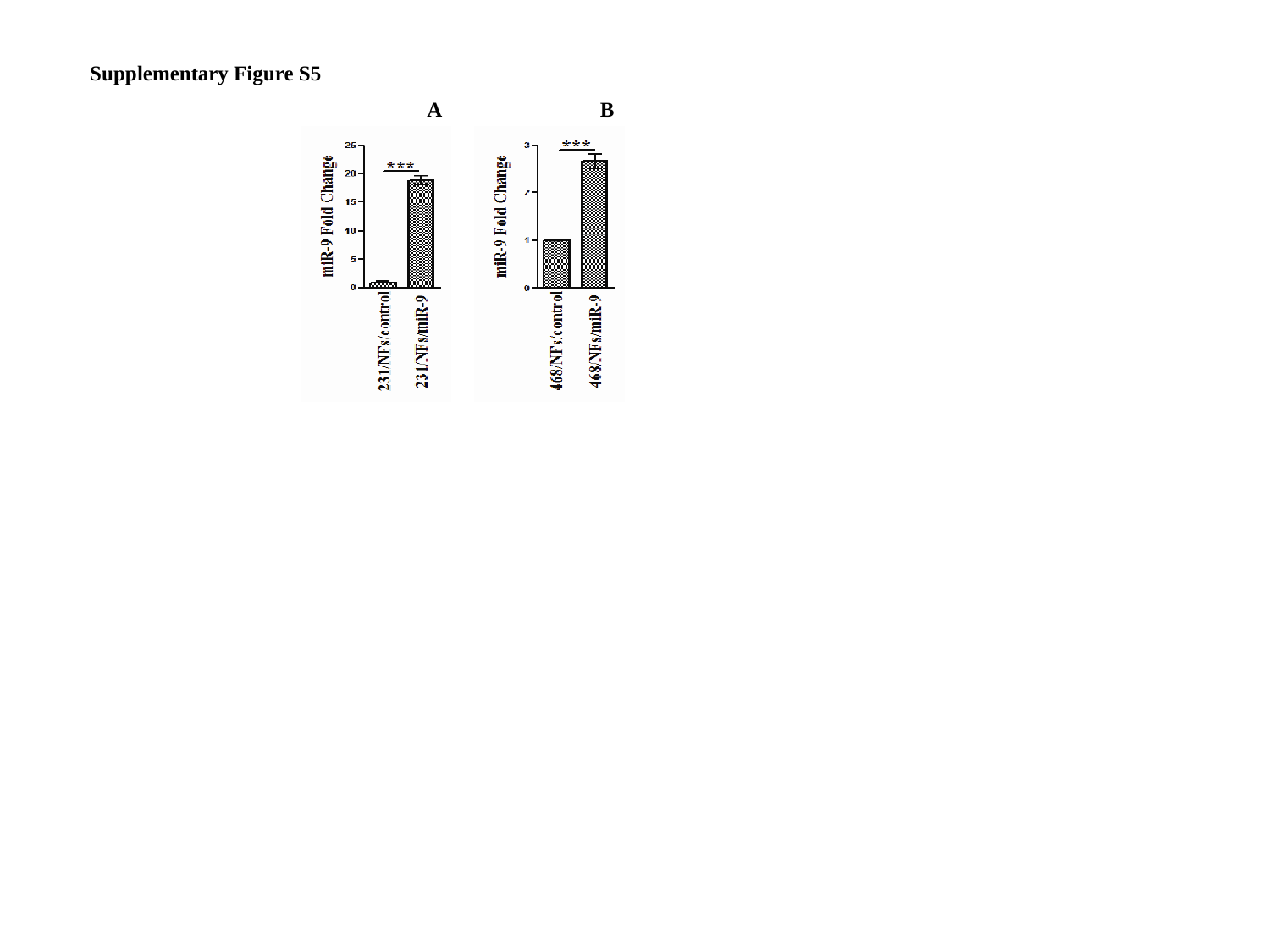

Supplementary Figure S5
A
B

Supplement: Supplementary Figure S5 [file cddis2016224x5.ppt]

## Slide 1
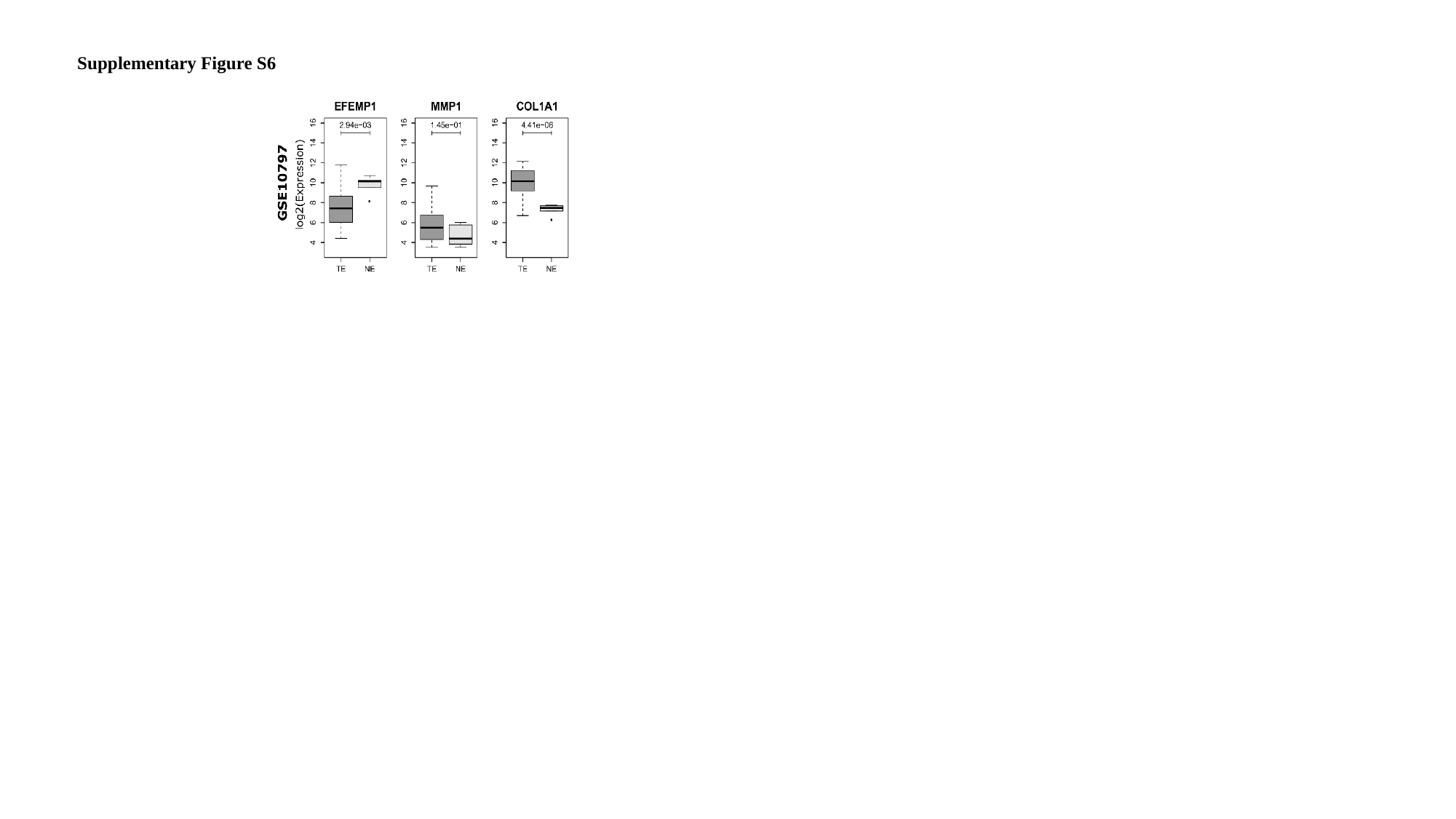

Supplementary Figure S6

Supplement: Supplementary Figure S6 [file cddis2016224x6.ppt]
