# Supplementary material for: Exosome-mediated delivery of miR-9 induces cancer-associated fibroblast-like properties in human breast fibroblasts
Source: Cell Death Dis. 2016 Jul 28;7(7):e2312–. doi: 10.1038/cddis.2016.224 (PMC4973361; doi:10.1038/cddis.2016.224)
Supplement: Supplementary Information [file cddis2016224x7.doc]

**Supplementary Figure Legends.**

**Supplementary Figure S1. Characterization of normal and cancer-associated fibroblasts.** αSMA protein expression evaluated by western blot analysis in CAFs compared to NFs. The lanes corresponding to the two samples of interest have been cropped from the same gel.

**Supplementary Figure S2. Biochemical characterization of breast cancer cell originated-exosomes.** Lamp2: lysosome-associated membrane protein 2, CD63: tetraspanin CD63 Flot1: flotillin 1, Rab5B: Ras-related protein Rab5B. Exo = exosomal extract, TE = total cell extract. Exosomes from MDA-MB-231 supernatant and MDA-MB-231 cells are characterized by western blot analysis for Lamp2, CD63, Flot1, Rab5B (exosome markers) compared with Vinculin and αTubulin (cell markers). The lanes corresponding to the two samples of interest have been cropped from the same gel.

**Supplementary Figure S3. *Mir-9* is delivered to NFs via exosomes.** qRT-PCR analysis of *miR-9* level in A) MDA-MB-468 transiently transfected with *miR-9* or control, B) exosomes purified from tumor cells and C) recipient NFs. Data are shown as normalized relative to *miR-21* (exosomes) or *RNU44* (MDA-MB-468 and NFs, respectively). (***p < 0.0005).

**Supplementary Figure S4. *Mir-9* internalized by fibroblasts specifically derived from cancer cell MDA-MB-231.** qRT-PCR analysis to assess *miR-9* level in A) MDA-MB-231 transiently transfected with *miR-9* or control, B) exosomes and C) NFs co-cultured with breast cancer cells derived-vesicles in exosome–deprivated medium (e-dm) compared with complete medium. Data are shown as normalized relative to *miR-21* (exosomes) or *RNU44* (MDA-MB-231 and NFs, respectively). D) qRT-PCR analysis to evaluate *miR-9* level in MDA-MB-231 transiently transfected with *miR-9* or control (left panel) and in NFs incubated with tumor-secreted exosomes (right panel) upon the additional step of medium change. E) Migration by transwell of recipient NFs grown in contact with breast cancer-derived exosomes. Quantitative analysis of the experiment is shown in the lower histograms. Data are presented as mean ± S.D. Scale bars, 100 µm. (*p < 0.05; **p < 0.005; ***p < 0.0005)

**Supplementary Figure S5. *Mir-9* released by microenvironment to neoplastic cells enhances tumor aggressiveness.** qRT-PCR analysis to evaluate *miR-9* uptake in A) MDA-MB-231 and B) MDA-MB-468 grown in contact with supernatant from NFs/miR-9 or NFs/control. (***p < 0.0005).

**Supplementary Figure S6.** **Gene expression analysis in public datasets.** Boxplots showing the expression levels of the 3 selected genes in a public gene expression dataset of breast tumor (TE) and normal epithelium (NE). P-values from two-tailed Student’s t-test are reported.
